# Bacteriome and Mycobiome Interactions Underscore Microbial Dysbiosis in Familial Crohn’s Disease

**DOI:** 10.1128/mBio.01250-16

**Published:** 2016-09-20

**Authors:** G. Hoarau, P. K. Mukherjee, C. Gower-Rousseau, C. Hager, J. Chandra, M. A. Retuerto, C. Neut, S. Vermeire, J. Clemente, J. F. Colombel, H. Fujioka, D. Poulain, B. Sendid, M. A. Ghannoum

**Affiliations:** a Inserm U995-Team 2, Université Lille 2, Faculté de Médecine H. Warembourg, Pôle Recherche, CHRU de Lille, Lille, France; b Center for Medical Mycology, University Hospitals Case Medical Center, Case Western Reserve University, Cleveland, Ohio, USA; c Epimad Registry, Epidemiology Unit and LIRIC Inserm 995, Lille University and Hospital, Lille, France; d Department of Gastroenterology, University Hospital Leuven, Leuven, Belgium; e Department of Genetics and Genomic Sciences, Icahn School of Medicine at Mount Sinai, New York, New York, USA; f Immunology Institute, Icahn School of Medicine at Mount Sinai, New York, New York, USA; g Department of Gastroenterology, Icahn School of Medicine at Mount Sinai, New York, New York, USA; h EM Core Facility, School of Medicine, Case Western Reserve University, Cleveland, Ohio, USA

## Abstract

Crohn’s disease (CD) results from a complex interplay between host genetic factors and endogenous microbial communities. In the current study, we used Ion Torrent sequencing to characterize the gut bacterial microbiota (bacteriome) and fungal community (mycobiome) in patients with CD and their nondiseased first-degree relatives (NCDR) in 9 familial clusters living in northern France-Belgium and in healthy individuals from 4 families living in the same area (non-CD unrelated [NCDU]). Principal component, diversity, and abundance analyses were conducted, and CD-associated inter- and intrakingdom microbial correlations were determined. Significant microbial interactions were identified and validated using single- and mixed-species biofilms. CD and NCDR groups clustered together in the mycobiome but not in the bacteriome. Microbiotas of familial (CD and NCDR) samples were distinct from those of nonfamilial (NCDU) samples. The abundance of *Serratia marcescens* and *Escherichia coli* was elevated in CD patients, while that of beneficial bacteria was decreased. The abundance of the fungus *Candida tropicalis* was significantly higher in CD than in NCDR (*P* = 0.003) samples and positively correlated with levels of anti-*Saccharomyces cerevisiae* antibodies (ASCA). The abundance of *C. tropicalis* was positively correlated with *S. marcescens* and *E. coli*, suggesting that these organisms interact in the gut. The mass and thickness of triple-species (*C. tropicalis* plus *S. marcescens* plus *E. coli*) biofilm were significantly greater than those of single- and double-species biofilms. *C. tropicalis* biofilms comprised blastospores, while double- and triple-species biofilms were enriched in hyphae. *S. marcescens* used fimbriae to coaggregate or attach with *C. tropicalis*/*E. coli*, while *E. coli* was closely apposed with *C. tropicalis*. Specific interkingdom microbial interactions may be key determinants in CD.

## IMPORTANCE

Here, we characterized the gut bacterial microbiota (bacteriome) and fungal community (mycobiome) in multiplex families with CD and healthy relatives and defined the microbial interactions leading to dysbiosis in CD. We identified fungal (*Candida tropicalis*) and bacterial (*Serratia marcescens* and *Escherichia coli*) species that are associated with CD dysbiosis. Additionally, we found that the level of anti-*Saccharomyces cerevisiae* antibodies (ASCA; a known CD biomarker) was associated with the abundance of *C. tropicalis*. We also identified positive interkingdom correlations between *C. tropicalis*, *E. coli*, and *S. marcescens* in CD patients and validated these correlations using *in vitro* biofilms. These results provide insight into the roles of bacteria and fungi in CD and may lead to the development of novel treatment approaches and diagnostic assays.

## INTRODUCTION

Crohn’s disease (CD) is a relapsing inflammatory bowel disease (IBD) that may affect many parts of the gastrointestinal (GI) tract and is driven by an abnormal immune response to gut microbial antigens, suggesting a complex interplay between host genetic factors and endogenous microbial communities. Recent studies have identified luminal bacterial species as associated with beneficial or deleterious effects. While most microbiome studies have focused on the bacterial community (bacteriome), it is only recently that sequencing-based investigations of the gut microbial community have started to pay some attention to the fungal community (mycobiome) (1–4). These studies concordantly revealed the importance of this neglected component of the microbiome and confirmed its involvement in *Candida*-host interplay in the setting of CD (5, 6). The composition of the intestinal microbiota is influenced by the genetic background of the host and other factors such as dietary habits and the environment. Both genetic and environmental factors are shared within families, and first-degree relatives of patients with CD are at much higher risk of developing CD than are the general population (7, 8).

The aim of the current study was to investigate to what extent the predominant fecal bacteriome and mycobiome of patients with familial CD have unique characteristics that distinguish them from those of healthy subjects. To reduce the confounding effect of genetics and environmental variables on interpretation of microbial dysbiosis in CD, we focused on patients with CD and their nondiseased relatives and included unrelated families of healthy individuals as controls. We used Ion Torrent sequencing to characterize the gut bacteriome and mycobiome in members of 9 families recruited in the north of France and Belgium where at least one patient had CD in comparison with their healthy relatives and members of 4 control families living in the same area.

The levels of anti-*Saccharomyces cerevisiae* antibodies (ASCA; a CD biomarker reported as being generated by *Candida*) were also determined. Our analysis identified bacterial and fungal species that are associated with CD dysbiosis and revealed positive interkingdom correlations between three species from fungal and bacterial communities in CD patients. To validate these correlations, we explored these interactions through biofilm formation, a mode of pathogenic development used by members of both kingdoms to reinforce their pathogenic potential as well as their ability to escape host defenses.

## RESULTS

### Patient demographics.

The current study analyzed fecal samples from 9 multiplex families comprising CD patients (*n* = 20) and their cohabiting non-CD relatives (NCDR; *n* = 28). Individuals from four unrelated healthy families with no history of CD (NCDU; *n* = 21) living in the same geographic area were used as comparators (participant demographics and clinical features of CD in the enrolled patients are summarized in Tables 1 and 2, respectively).

**TABLE 1:** Demographics of enrolled study participants

Characteristic	CD	NCDR	NCDU
No. of			
Families	9	9	4
Individuals	20	28	21
Females	12	13	13
Males	8	15	8
Age (mean, yr)	44.5	48.4	41.3

**TABLE 2:** Clinical features of CD patients^a^

Variable	CD characteristic	Frequency
Age category	A1 (≤16 yr)	0
	A2 (17–40 yr)	8
	A3 (≥40 yr)	12
Location	L1 (terminal ileum)	11
	L2 (colon)	2
	L3 (ileum-colon)	6
	L4 (upper GI tract)	0
Behavior	B1 (nonstenotic)	3
	B2 (stenotic)	4
	B3 (penetrating)	12
Disease status	Active	3
	Remission	8

a Data collected at sampling time.

### Microbiotas of familial samples are distinct from those of nonfamilial samples.

**FIG 1:**
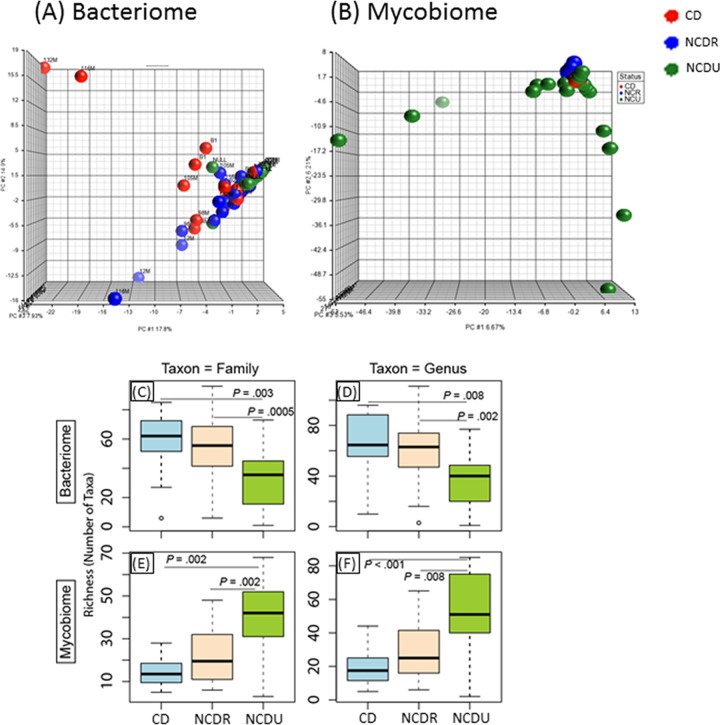
Distribution of bacteriome and mycobiome in enrolled individuals. (A and B) Clustering of genera in bacteriome (A) and mycobiome (B) in Crohn’s disease (CD), non-Crohn’s disease relative (NCDR), and non-Crohn’s disease unrelated individual (NCDU) groups. (C to F) Richness of microbiota in bacteriome (C and D) and mycobiome (E and F) at fungal family, genus, and species taxon levels.

Principal component analysis (PCA) showed that for the bacteriome, CD, NCDR, and NCDU samples were widely scattered (Fig. 1A). In contrast, for the mycobiome this scattering was limited to NCDU while CD and NCDR clustered together (Fig. 1B). The richness of the bacteriome in CD and NCDR samples was significantly higher than that in the NCDU group (Fig. 1C and D). Interestingly, an opposite pattern was observed for the mycobiome, with significantly increased richness in the NCDU group compared to the CD or NCDR group (Fig. 1E and F). No difference in the richness of the mycobiome was noted in samples collected from CD patients and their healthy relatives (NCDR). These data demonstrate that samples from related individuals have greater similarity to each other irrespective of their CD status. Therefore, comparison of the microbiotas within affected and unaffected family members may provide insights on organisms on dysbiosis linked to disease. Thus, in subsequent analyses we performed comparisons between CD patients and their healthy, non-CD relatives (NCDR).

### Abundance of potentially pathogenic bacteria is increased while beneficial bacteria are decreased in CD.

**FIG 2:**
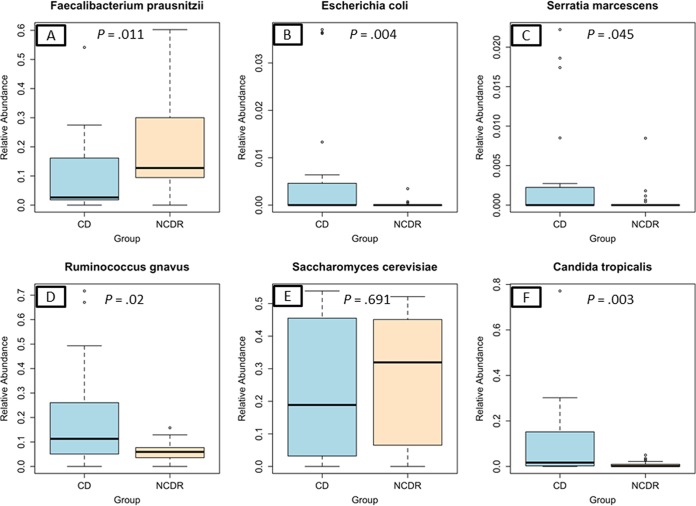
Abundance of CD-associated bacterial and fungal species in study participants. (A) *Faecalibacterium prausnitzii*. (B) *Escherichia coli*. (C) *Serratia marcescens*. (D) *Ruminococcus gnavus*. (E) *Saccharomyces cerevisiae*. (F) *Candida tropicalis*.

Analyses of the abundance of bacterial and fungal communities revealed the presence of five and four phyla, respectively, with >1% abundance. The most abundant bacterial phylum was *Firmicutes* (median abundance, ~68%) followed by *Actinobacteria* (12.6% to 17.96%) and *Proteobacteria* (1.9% to 2.4%) or *Bacteroidetes* (0.9% to 7.9%) (see Table S2 in the supplemental material). Interestingly, levels of *Bacteroidetes* were significantly reduced in CD patients compared to NCDR (0.9% and 7.8%, respectively; *P* = 0.001). This decrease of *Bacteroidetes* in CD patients was consistently observed at other taxon levels of this phylum (see Tables S3 and S4). *Bifidobacterium adolescentis* and *Ruminococcus gnavus* were the most abundant bacterial species in the CD group (19.8% and 19.1%, respectively), while in the NCDR group the most abundant bacterial species were *Bifidobacterium adolescentis* and *Faecalibacterium prausnitzii* (20% and 19%, respectively; see Table S4). Abundances of 11 genera and 15 species differed significantly between CD and NCDR groups. These included an increase in the abundance of potentially pathogenic bacterial species like *Escherichia coli* (*P* = 0.004), *Serratia marcescens* (*P* = 0.045), and *Ruminococcus gnavus* (*P* = 0.02) (Fig. 2). In contrast, the abundance of *Faecalibacterium prausnitzii* was elevated in NCDR compared to CD patients (*P* = 0.034) (Fig. 2).

### *Candida tropicalis* abundance is significantly increased in CD patients.

Mycobiome analysis showed that Ascomycota and Basidiomycota were the two phyla present at >1% abundance, with Ascomycota being the most abundant in both CD and NCDR groups (≥74%) (see Table S5 in the supplemental material). Comparison of the abundance of different taxon levels (class through genus) showed no significant differences between CD and NCDR groups (see Tables S6 and S7). *Saccharomyces cerevisiae* and *Candida tropicalis* were the most common known fungal species in the CD group (24% and 10%, respectively), while *Saccharomyces cerevisiae* and *Galactomyces geotrichum* (27% and 8%, respectively) were the most abundant in the NCDR group (see Table S8). However, the abundance of the nonpathogenic yeast *Saccharomyces cerevisiae* tended to increase in healthy (NCDR) individuals (*P* = 0.691) (Fig. 2E), while one fungus (*Candida tropicalis*) exhibited a significant difference in abundance between CD and NCDR groups (10.41% versus 0.79%, respectively, *P* = 0.003) (Fig. 2F).

Since yeasts of the genus *Candida* have been described as immunogens for CD biomarkers designated anti-*Saccharomyces cerevisiae* antibodies (ASCA) (9, 10), we investigated correlations between *C. tropicalis* abundance and ASCA levels. Our data showed that the ASCA level was significantly higher in the CD than in the NCDR group (*P* = 0.001) and that *C. tropicalis* was the only fungus that was positively associated with ASCA (*P* ≤ 0.001). No significant association was found between the abundance of *Candida* spp., including *C. tropicalis*, and other CD variables, including age at diagnosis, location, behavior, or NOD2 polymorphisms (data not shown).

### CD is associated with inter- and intrakingdom correlations.

**FIG 3:**
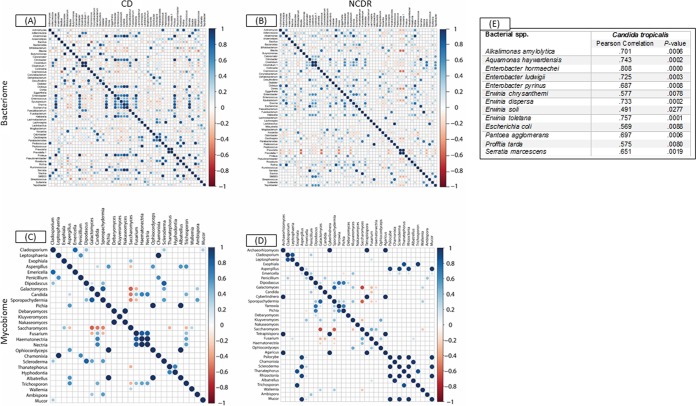
Associations among bacterial and fungal genera in CD patients (A and C) and their cohabiting non-CD relatives (B and D). (A and B) Bacteriome. (C and D) Mycobiome. Red circles indicate negative associations, while blue circles indicate positive associations. Diameters of circles indicate the magnitude of the correlation (−1 through +1) for each fungal pair. Only significant associations (*P* < 0.05) are shown.

Next, we performed unbiased correlation analyses to explore the relationship among and between the members of the gut microbiota in the setting of CD. Our analyses revealed several significant associations at the genus and species levels in both bacteria and fungi (Fig. 3A to D; also see Tables S9 and S10 in the supplemental material). At the genus level, there were 562 intrakingdom correlations in the bacteriome (270 in CD and 292 in NCDR) and 272 correlations within the mycobiome (124 in CD and 148 in NCDR). *Candida* exhibited 6 significant intrakingdom correlations with known fungal genera, of which five were positive (*Fusarium*, *Haematonectria*, *Nectria*, *Thanatephorus*, and *Trichosporon*) while one was negative (association with *Saccharomyces*), which confirmed the results gained from the abundance study. In addition, significant interkingdom associations were detected, including six bacterial-fungal genus correlations (Table 3). At the species level, *C. tropicalis* exhibited significantly positive associations with 13 bacterial species, including *E. coli* and S. marcescens (Fig. 3E).

**TABLE 3:** Significant correlations between bacterial and fungal genera

Bacterial genus	Fungal genus	Pearson correlation	*P* value
*Faecalibacterium*	*Kluyveromyces*	0.520	0.019
*Prevotella*	*Kluyveromyces*	0.980	<0.001
*Oscillospira*	*Pichia*	0.724	<0.002
*Oscillospira*	*Ophiocordyceps*	0.717	<0.003
*Oscillospira*	*Albatrellus*	0.717	<0.004
*Proteus*	*Candida*	0.709	<0.005

### Biofilm formation mediates interkingdom interactions in CD.

**FIG 4:**
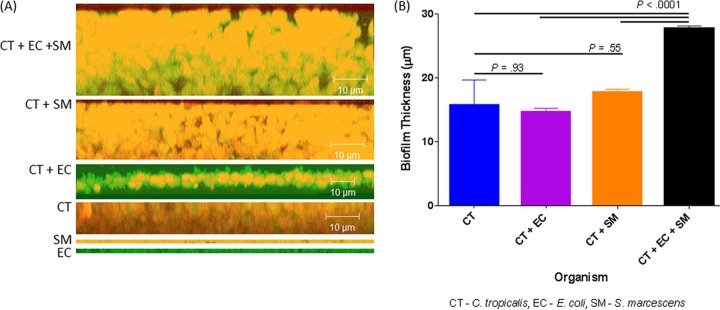
Confocal analysis of biofilms formed by *C. tropicalis* (CT) alone or in combination with *E. coli* (EC) and/or *S. marcescens* (SM). (A) Side view of biofilms formed by *C. tropicalis* plus *E. coli* plus *S. marcescens*, *C. tropicalis* plus *S. marcescens*, *C. tropicalis* plus *E. coli*, *C. tropicalis* alone, *S. marcescens* alone, or *E. coli* alone. (B) Mean thickness of biofilms.

**FIG 5:**
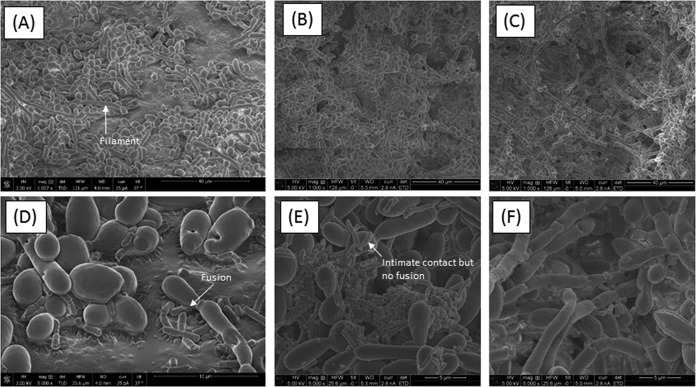
Scanning electron microscopy analyses of biofilms formed by *C. tropicalis* alone or in combination with *E. coli* and/or *S. marcescens*. (A) *C. tropicalis* plus *E. coli* (magnification, ×1,057); (B) *C. tropicalis* plus *S. marcescens* (magnification, ×1,000); (C) *C. tropicalis* plus *E. coli* plus *S. marcescens* (magnification, ×1,000); (D) *C. tropicalis* plus *E. coli* (magnification, ×5,000); (E) *C. tropicalis* plus *S. marcescens* (magnification, ×5,000); (F) *C. tropicalis* plus *E. coli* plus *S. marcescens* (magnification, ×5,000).

**FIG 6:**
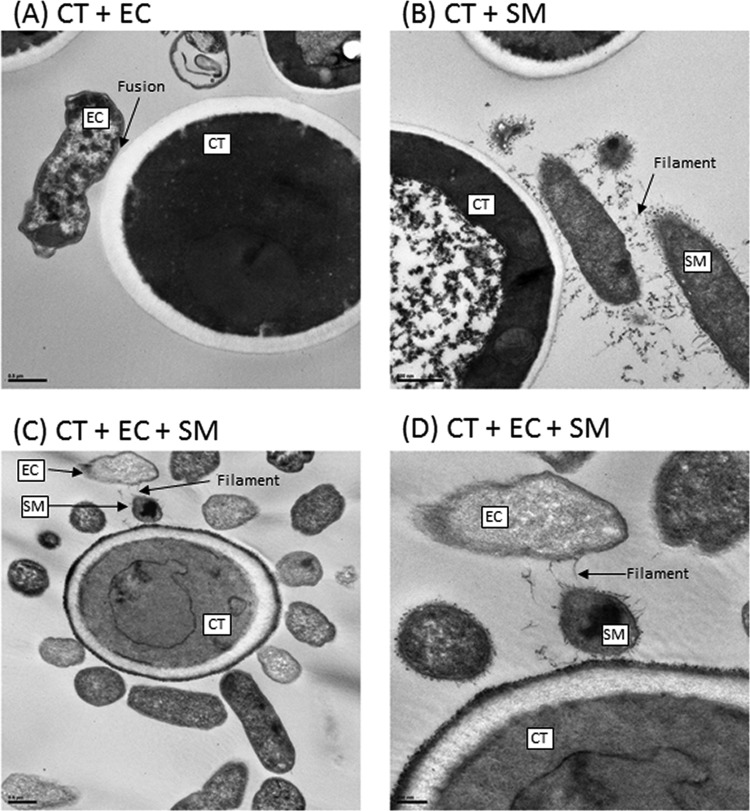
Transmission electron microscopy analyses of biofilms formed by *C. tropicalis* (CT) alone or in combination with *E. coli* (EC) and/or *S. marcescens* (SM). (A) *C. tropicalis* plus *E. coli* (bar, 0.5 µm); (B) *C. tropicalis* plus *S. marcescens* (bar, 500 nm); (C) *C. tropicalis* plus *E. coli* plus *S. marcescens* (bar, 0.5 µm); (D) *C. tropicalis* plus *E. coli* plus *S. marcescens* (bar, 200 nm).

The microbiome is likely a platform supporting a wide range of extremely complex molecular interactions and signal transductions that drive cooperation or antagonism among the microbial communities. Since *E. coli* and *S. marcescens* have been shown to interact with *C. tropicalis* (11, 12), we investigated their ability to form biofilms using our *in vitro* model (13). Confocal analyses showed that all the tested organisms were able to form biofilms, and the thickness of the triple-species biofilms was significantly greater (*P* < 0.0001) than that of biofilms formed by single and double species (Fig. 4). Scanning electron microscopy (SEM) analyses showed that while biofilms formed by *C. tropicalis* alone comprised yeast forms, those formed by *C. tropicalis* combined with either *E. coli* or *S. marcescens* were enriched in fungal hyphae, a form of growth associated with pathogenic conditions (Fig. 5A to C). Closer examination of these biofilms showed that the two bacteria existed in intimate contact with the fungus but differed in their specific interactions. In this regard, unlike *S. marcescens*, *E. coli* cells seem to be fused to the fungal cells (Fig. 5D and E). Further analyses using transmission electron microscopy (TEM) confirmed the findings of SEM, showing the close interactions of *C. tropicalis* with *E. coli* and/or *S. marcescens*. We found that *E. coli* cells were closely apposed with *C. tropicalis* (Fig. 6A), while *S. marcescens* cells produced fimbriae (diameter range, 3 to 18 nm; length range, 34 to 480 nm) that mediated attachment with *C. tropicalis* (Fig. 6B). Interestingly, in biofilms formed by the three organisms, *S. marcescens* cells interacted with both *C. tropicalis* and *E. coli* through these fimbriae (Fig. 6C and D).

## DISCUSSION

In this study, analysis of the gut bacteriome and mycobiome of CD patients in multiplex families compared to those of their unaffected first-degree relatives showed for the first time that interactions between endogenous gut bacteria and fungi are closely associated with human disease. Among hundreds of bacterial and fungal species residing in the gut, large-scale sequencing and bioinformatics unambiguously identified the association between a fungal species (*C. tropicalis*) and two bacterial species (*S. marcescens* and *E. coli*). We confirmed these interkingdom interactions among the three organisms when grown as mixed-species biofilms.

Our analyses of the diversity of gut microbial communities in CD, NCDR, and NCDU samples clearly revealed that the gut microbiotas of both CD patients and their first-degree healthy relatives were distinct from those of the unrelated healthy individuals. Our findings are in agreement with those of Joossens et al. (5), who used denaturing gradient gel electrophoresis (DGGE) and reported that bacterial microbiotas of unaffected relatives of patients with CD were different from those of healthy controls. Moreover, Schloss et al. (14) showed that members of a family share genetics, environment, diet, and bacterial microbiota and that the family members are more similar to each other than they are to unrelated individuals.

Our results demonstrate significant changes in bacterial and fungal taxa between the CD and NCDR groups. Among the bacteria, organisms belonging to the phylum *Bacteroidetes* were consistently reduced in CD patients, a finding which agrees with previous studies (1, 2, 15–17). We also found that levels of *E. coli*, *S. marcescens*, *Cronobacter sakazakii*, and *Ruminococcus gnavus* were significantly increased in CD while that of *F. prausnitzii* was significantly decreased in this group, compared to the NCDR controls. Previous studies have associated an increase in *E. coli* and a decrease in *F. prausnitzii* numbers with inflammatory bowel disease (IBD) (15), and the ratio of *F. prausnitzii* to *E. coli* bacteria has been proposed as an indicator of dysbiosis in CD patients. *C. sakazakii* is known to induce an increase in proinflammatory cytokines linked to increased oxidative damage and apoptotic cell death, followed by tissue damage and lesion formation in the gut epithelium (18). Separate studies have shown that *R. gnavus* produces mucolytic enzymes that can degrade the protective mucin layer of the gut epithelium, contributing to lesion formation (5, 19). Our study is the first to expand the microbial panel associated with bacterial dysbiosis in CD patients to include *S. marcescens*. In a study by Ochieng et al. (20), *S. marcescens* was shown to interact with intestinal epithelial cells in culture and induce dramatic immunological alterations similar to those produced by known enteric pathogens. Therefore, *S. marcescens* may have a critical role in CD by aggravating the inflammatory episodes.

Interestingly, *C. tropicalis* was the only fungal species significantly increased in abundance in CD patients compared to their non-CD relatives and controls and also was positively associated with ASCA (directed against terminal α-1,3-mannoside residues), a known biomarker of CD (21, 22). ASCA are antibodies directed against di- or tri-α-1-2-linked mannosides with an α-1,3 mannose at the nonreducing end (23, 24). Initial development of an ASCA-based test employed *S. cerevisiae* mannan, but subsequent studies have shown that any fungus that can produce these mannans will be detected using ASCA. For example, both rabbit and murine experimental models demonstrated that ASCA are also generated by *C. albicans* under pathogenic conditions and DSS-induced colitis (10, 25). Similar findings have been reported by Iliev et al. (26), who examined whether gut fungi can be detected by the immune system upon intestinal insult and found that intestinal inflammation led to the development of circulating ASCA, triggered by fungal antigens indigenous to the gut. Interestingly, 97.3% of all the fungal sequences identified from mouse stools belonged to 10 fungal species, with 65.2% of the sequences belonging to a single fungus, *Candida tropicalis*. The correlation observed in our study between ASCA and *C. tropicalis* confirms the notion that ASCA are pan-fungal antibodies and that the increase in ASCA in our patient cohort is at least in part due to an increase in *C. tropicalis* levels.

In a recent study, Whibley et al. (27) showed that caspase recruitment domain family member 9 (CARD9; a susceptibility gene for IBD) and tumor necrosis factor alpha (TNF-α) are involved in protection against systemic *C. tropicalis* infection, mediated by increased fungicidal activity of neutrophils. CARD9 is also known to be involved in the immune response against microorganisms and was recently shown to have a protective effect in colitis by modulating the microbial metabolism of tryptophan (28). Since *C. tropicalis* is also known to specifically interact with immune pathways involving CARD9 (27), it is possible that both bacterial and fungal communities modulate metabolic and host immune response pathways, exacerbating the disease in CD patients.

In our study, correlation analyses identified significant intra- as well as interkingdom associations in the bacteriome and mycobiome of CD patients, including at the species level, where *C. tropicalis* exhibited significant positive association with 13 bacterial species, including *E. coli* and *S. marcescens*. Similar interkingdom associations of microbiome in CD were recently reported by Sokol et al. (6), who showed that fungal genera (mostly *Saccharomyces* and *Malassezia*) were positively correlated with several bacterial taxa in CD, while no correlations were reported between *Candida* and bacteria. In contrast, our results showed *Saccharomyces* to be negatively correlated with most of the bacterial genera and identified significant correlations between *C. tropicalis* and potentially pathogenic bacteria. The differences between our study and that of Sokol et al. (6) could be attributed to the fact that we compared the mycobiome and bacteriome among genetically related individuals, while Sokol et al. (6) compared IBD patients with unrelated healthy subjects.

Biofilms (as is the situation in the GI tract) render the organisms resistant to antimicrobial agents and protect them from immune cells (29–31). Our *in vitro* studies demonstrate that *C. tropicalis*, *E. coli*, and *S. marcescens* cooperate to form robust biofilms comprising fungal hyphae and species-specific interactions. Fungal filamentation is a known virulence factor used by *Candida* to damage host tissues and to trigger specific host immune responses (32–35). Moreover, interactions between *C. tropicalis* and these two bacteria have been previously reported where lipopolysaccharide produced by *S. marcescens* and *E. coli* significantly enhanced fungal biofilm maturation (36, 37) Distinct interspecies interactions in this biofilm environment were clearly evident, where *E. coli* tended to be closely apposed with the fungal cell walls, while *S. marcescens* used its fimbriae to form a “bridge” between *C. tropicalis* and *E. coli*. Interactions between *S. marcescens* and eukaryotic cells mediated by d-mannose-recognizing pili have been shown earlier by Castro et al. (38) in insect guts. The molecular mechanisms underlying these interactions and their role in CD are currently being investigated.

Based on these findings, we propose that inter- and intrakingdom interactions impact the host immune system in the setting of CD. In Crohn’s disease, levels of proinflammatory cytokines (e.g., Th17 cytokines) may increase under the influence of enteric pathogens and immunomodulatory components of biofilms (e.g., fungal β-glucans and bacterial lipopolysaccharides), causing increased oxidative damage and apoptotic cell death. Additionally, microbe-induced production of mucolytic enzymes may lead to barrier dysfunction, resulting in tissue damage and lesion formation.

Taken together, our results suggest that *C. tropicalis* interacts with potential bacterial pathogens and that these interactions may play an important role in CD.

## MATERIALS AND METHODS

### Study cohorts.

We analyzed the intrafamilial distribution of the bacteriome and mycobiome in 13 families recorded through the population-based EPIMAD registry (France) (39–41) and the Inflammatory Bowel Disease Registry at the University Hospital, Gasthuisberg, Leuven (Belgium). These were distributed in 9 multiple affected families with at least 3 first-degree relatives with CD and 4 healthy control unrelated families (Table 1) (22). The control families were recruited in France, and they belonged to the same generation, had equal compositions of males and females, and consisted of a comparable number of persons within the family. All participants gave stool and blood samples after written informed consent. Medical records of all affected members of the families were reviewed by independent gastroenterologists from 2 different university hospitals, according to the methodology of the EPIMAD registry (40). Families were interviewed and samples were collected in their homes during meetings that were attended by both affected and unaffected members. Among the 9 multiplex CD families, 67% of patients were living together in the same household (*n* = 36) at the time of interview and sample collection. The study was approved by the Ethics Committee of the Catholic University of Leuven and by the CCPPRB of Lille (reference no. CP 00/60, year 2000).

We characterized the bacteriome and mycobiome in multiplex families comprising CD patients (*n* = 20) and their cohabiting non-CD first-degree relatives exposed to the same environmental factors (NCDR, *n* = 28). Four unrelated healthy families with no history of CD (NCDU, *n* = 21) living in the same geographic area were used as comparators (participant demographics and clinical features of CD in the enrolled patients are summarized in Table S1 in the supplemental material).

The following information was recorded: age at diagnosis, gender, date of CD diagnosis, smoking status, extraintestinal manifestations (joint, skin, ocular, and hepatobiliary manifestations), and details of family history of disease. In addition, disease location and behavior according to the Montreal classification (42, 43) and treatments (systemic steroids, immunosuppressive therapy, and biotherapy) received during the follow-up, including intestinal resection (date and type), were retrospectively collected at diagnosis and during maximal follow-up. In the Montreal classification, A1 includes CD patients diagnosed at an age of <17 years, A2 includes those diagnosed from 17 to 39 years, and A3 includes those diagnosed at >40 years of age. The following classifications regarding CD location were captured: L1, pure ileal disease; L2, pure colonic disease; L3, ileocolonic disease (L1 with cecal involvement was considered L3); and L4, upper gastrointestinal disease (which could be associated with L1, L2, or L3). Perianal lesions were also recorded. CD behavior was classified as B1 (inflammatory), B2 (structuring), and B3 (penetrating). The B2 and B3 classifications were considered “complicated behavior.”

### Collection, transfer, and storage of fecal samples.

In each center, written informed consent was obtained from each participant after a full explanation of the study. Crohn’s disease patients and controls were asked to collect the stool samples from the first bowel movement in the morning. Stool samples were collected by using a specific kit. It consists of a ready-to-use package, including a user guide and a sterile liquid-absorbing plastic bag to be placed across the rim of the toilet. Stool samples ware homogenized, inserted in sterile containers (Sarstedt, Germany) using a collection spoon, and immediately frozen at −30°C. Collected samples were transported frozen over dry ice as one batch to Cleveland, OH, USA, where they were kept at −80°C until processing.

### Detection of anti-*Saccharomyces cerevisiae* antibodies.

An enzyme-linked immunosorbent assay (ELISA) was used to detect ASCA. ASCA (immunoglobulin G, A, and M) titers were expressed in arbitrary units (AU) according to a calibration curve established for each experiment as described previously (22).

### DNA extraction.

Fungal and bacterial genomic DNAs were isolated and purified with the QIAamp DNA stool minikit (Qiagen) according to the manufacturer’s instructions with minor modifications. Briefly, 3 additional bead-beating steps (Sigma-Aldrich beads; diameter, 500 µm) with the MP Fastprep-24 (speed setting of 6, 3 runs of 60 s) after the stool lysis step (in ASL buffer) were performed. The quality and purity of the isolated genomic DNA were confirmed spectrophotometrically using a NanoDrop 2000 device (Fisher Scientific SAS, Illkirch, France). DNA concentration was quantified using the Qubit 2.0 instrument applying the Qubit double-stranded DNA (dsDNA) HS assay (Life Technologies, USA). Extracted DNA samples were stored at −20°C.

### Microbiome analyses.

Analysis of the microbiome profile in the extracted DNA samples was conducted as described previously by our group (44, 45). A brief summary of the method is provided below.

### (i) Amplicon library preparation.

The internal transcribed spacer 1 (ITS1) and 16S rRNA gene regions for fungi and bacteria, respectively, were amplified as described previously (45). Briefly, the ITS1 region was amplified using ITS1F (CTTGGTCATTTAGAGGAAGTAA) and ITS2 (GCTGCGTTCTTCATCGATGC) primers. The reactions were carried out on 100-ng template DNA, in a 50-µl (final volume) reaction mixture consisting of Dream Taq Green PCR master mix (Thermo Scientific), 0.1 g/liter bovine serum albumin, 1% dimethyl sulfoxide (DMSO), 6 mM MgCl_2_, and a final primer concentration of 400 nM. Initial denaturation at 94°C for 3 min was followed by 35 cycles of denaturation for 30 s each at 94°C, annealing at 50°C for 30 s, and extension at 72°C for 1 min. Following the 35 cycles, there was a final extension time of 5 min at 72°C. The V4 region of the 16S rRNA gene was amplified using 16S-515F (GTGCCAGCMGCCGCGGTAA) and 16S-806R (GGACTACHVGGGTWTCTAAT) primers. The reactions were carried out on 100-ng template DNA, in a 50-µl (final volume) reaction mixture consisting of Dream Taq Green PCR master mix (Thermo Scientific), 0.1 g/liter bovine serum albumin, 1% dimethyl sulfoxide (DMSO), 6 mM MgCl_2_, and a final primer concentration of 400 nM. Initial denaturation at 94°C for 3 min was followed by 30 cycles of denaturation for 30 s each at 94°C, annealing at 50°C for 30 s, and extension at 72°C for 1 min. Following the 30 cycles, there was a final extension time of 5 min at 72°C. The size and quality of amplicons were screened by 1.5% Tris-acetate-EDTA agarose gel electrophoresis, using 100 V; the gels were electrophoresed for 45 min and stained with ethidium bromide.

The PCR products were sheared for 20 min, using the Ion Shear Plus fragment library kit (Life Technologies, NY, USA). The amplicon library was generated with sheared PCR products using Ion Plus fragment library kits (<350 bp) according to the manufacturer’s instructions. The library was barcoded with the Ion Xpress barcode adapter and ligated with the A and P1 adapters.

### (ii) Sequencing, classification, and analysis.

The adapted barcoded libraries were equalized using the Ion library equalizer kit to a final concentration of 100 pM. Once equalized, the samples were pooled, diluted to 26 pM, and attached to the surface of Ion Sphere particles (ISPs) using an Ion PGM Template OT2 200-bp kit v2 (Life Technologies, USA) according to the manufacturer’s instructions, via emulsion PCR. The quality of ISP templates was checked using an Ion Sphere quality control kit (catalog no. 4468656) with the Qubit 2.0 device. Sequencing of the pooled libraries was carried out on the Ion Torrent Personal Genome Machine (PGM) system using the Ion Sequencing 200 kit v2 (all from Life Technologies) for 150 cycles (600 flows), with a 318 chip according to the manufacturer’s instructions. Demultiplexing and classification were performed using the Qiime 1.6 platform. The resulting sequence data were trimmed to remove adapters, barcodes, and primers during the demultiplexing process. In addition, the bioinformatics process filters were applied to the sequence data for the removal of low-quality reads with Phred scores of below Q25 and denoised to exclude sequences with read lengths of less than 100 bp (46). *De novo* operational taxonomic units (OTUs) were clustered using the Uclust algorithm and defined by 97% sequence similarity (47). Classification at the species level was referenced using the UNITE 5.8S database, and taxa were assigned using the nBlast method with a 90% confidence cutoff (48, 49). Abundance profiles for the bacteriome and mycobiome were generated and imported into Partek Discover Suite (v6.11) for principal component analysis (PCA).

### Bioinformatics and statistical analyses.

The statistical programming language R and related packages (50) were used for diversity and correlation analyses and Kruskal-Wallis (nonparametric) analysis of variance using abundance data. Diversity was analyzed using the Shannon diversity index (which characterizes species diversity) and richness (number of organisms in a sample) at all taxonomic levels using the R package *vegan* (51). All groupwise comparisons were conducted with SPSS (ver. 22), and a *P* value of <0.05 was considered statistically significant.

### Biofilm formation.

Biofilms were formed using *C. tropicalis*, *S. marcescens*, or *E. coli* singly or in double- or triple-species combinations and analyzed using metabolic activity assays, confocal microscopy, scanning electron microscopy, and transmission electron microscopy as described previously (13). Experiments were performed in triplicate, and mean values ± standard deviations (SD) were reported for quantitative results.

## SUPPLEMENTAL MATERIAL

Table S1  Abundance of bacterial phyla, classes, and orders in CD and NCDR groups. Table S1, XLSX file, 0.02 MB

Table S2  Abundance of bacterial families in CD and NCDR groups. Table S2, XLSX file, 0.02 MB

Table S3  Abundance of bacterial genera in CD and NCDR groups. Table S3, XLSX file, 0.02 MB

Table S4  Abundance of bacterial species in CD and NCDR groups. Table S4, XLSX file, 0.02 MB

Table S5  Abundance of fungal phyla, classes, and orders in CD and NCDR groups. Table S5, XLSX file, 0.02 MB

Table S6  Abundance of fungal families in CD and NCDR groups. Table S6, XLSX file, 0.02 MB

Table S7  Abundance of fungal genera in CD and NCDR groups. Table S7, XLSX file, 0.02 MB

Table S8  Abundance of fungal species in CD and NCDR groups. Table S8, XLSX file, 0.02 MB

Table S9  Intra- and interkingdom correlations at the genus level. Table S9, XLSX file, 1.1 MB

Table S10  Intra- and interkingdom correlations at the species level. Table S10, XLSX file, 2 MB
